# Improving object detection in challenging weather for autonomous driving via adversarial image translation

**DOI:** 10.1371/journal.pone.0333928

**Published:** 2025-10-14

**Authors:** Kunyi Wang, Yaohua Zhao

**Affiliations:** 1 University of British Columbia, Vancouver, BC, Canada; 2 School of Trafic & Transportation Engineering, Central South University, Changsha, China; Guangdong University of Petrochemical Technology, CHINA

## Abstract

Vision-based environmental perception is fundamental to autonomous driving, as it enables reliable detection and recognition of diverse objects in complex traffic environments. However, adverse weather conditions (such as rain, fog, and low-light conditions) significantly degrade image quality, thereby undermining the reliability of object detection algorithms. To address this challenge, we propose a two-stage framework designed to enhance object detection under adverse conditions. In the first stage, we design a lightweight Pix2Pix-based generative adversarial network (LP-GAN) that translates adverse-weather images into clear-weather counterparts, thereby alleviating visual degradation. In the second stage, the translated images are processed by a state-of-the-art object detector (YOLOv8) to enhance robustness and accuracy. Extensive experiments on the CARLA simulator demonstrate that the proposed framework substantially improves detection performance across diverse adverse conditions. Furthermore, the generated clear-weather images provide faithful and interpretable visual representations, which can facilitate human understanding and decision-making in autonomous driving. Overall, the proposed framework offers a practical and effective solution for weather-robust object detection, thereby contributing to safer and more reliable autonomous driving.

## 1. Introduction

In recent years, autonomous driving has emerged as a transformative paradigm that is reshaping the future of transportation, with the potential to deliver unprecedented advances in safety, efficiency, and accessibility [1–3]. At the core of this paradigm is a holistic architecture encompassing perception, planning, decision-making, and control. Among these, perception serves as the cornerstone, enabling the vehicle to construct a real-time and structured representation of its complex, dynamic surroundings [4]. Vision-based environmental perception [5–8], in particular, has become indispensable due to its rich semantic information and cost-effectiveness, underpinning a broad spectrum of downstream tasks. Central to this perceptual pipeline is object detection, a critical capability that facilitates the identification and localization of surrounding agents and infrastructure elements. As the principal mechanism by which autonomous systems interpret the visual world, object detection transcends a functional role and constitutes a safety-critical technology. It ensures collision avoidance, trajectory planning, and regulation-compliant navigation across diverse traffic scenarios [9]. Therefore, its reliability is fundamental to large-scale deployment and societal acceptance of autonomous driving.

Adverse weather remains one of the most critical barriers to the safe deployment of autonomous vehicles. Numerous real-world incidents have underscored the vulnerability of perception systems when confronted with degraded visibility [10–12]. For example, heavy rain can create specular reflections and water spray that obscure lane markings, while dense fog reduces contrast and blurs distant objects, and nighttime illumination often results in sensor noise and incomplete detection of pedestrians or obstacles. Such environmental factors directly impair core perception modules (i.e., object detection, lane keeping, and semantic segmentation), leading to cascading failures in planning and control. While state-of-the-art vision-based models achieve near-human performance under clear-weather conditions, their accuracy deteriorates sharply in adverse environments, raising serious concerns about the reliability of autonomous systems in safety-critical scenarios. Motivated by these challenges, this work focuses on enhancing object detection robustness under adverse weather.

Driven by the extraordinary success of deep learning [13], object detection has experienced significant evolution over the past decade, transitioning from early region-based approaches to highly efficient, end-to-end trainable frameworks. Notable milestones include the introduction of the two-stage Region-based Convolutional Neural Network (R-CNN) family [14], which markedly advanced detection accuracy. This was followed by the development of one-stage detectors such as the You Only Look Once (YOLO) series [15] and the Single Shot MultiBox Detector (SSD) [16], which attained real-time performance while maintaining competitive accuracy. Recent advances, including YOLOv5, YOLOv8 and Faster R-CNN with feature pyramid networks (FPN) [17], have further elevated the performance of object detectors under standard conditions, making them integral components of modern autonomous driving perception stacks. Despite their impressive capabilities, these models are predominantly trained and evaluated on datasets collected under clear-weather, daylight conditions, which limits their robustness in real-world environments characterized by adverse weather and low-illumination scenarios. Rain, fog, snow, and nighttime lighting can severely degrade image quality, introduce visual artifacts, and obscure critical scene elements, resulting in pronounced degradation of detection accuracy [18,19]. This vulnerability poses a significant challenge to the deployment of autonomous vehicles in all-weather, all-day conditions and underscores the urgent need for perception systems that are resilient to environmental variability.

To address the vulnerability of object detection models under adverse environmental conditions, we propose a novel two-stage framework designed to enhance robust perception in autonomous driving scenarios. In the first stage, a lightweight Pix2Pix-based generative adversarial network (LP-GAN) is employed for image-to-image translation, reconstructing clear-weather visual features from degraded inputs captured under rain, fog, and nighttime scenarios. In the second stage, the translated images are processed by YOLOv5, yielding substantial gains in detection accuracy and robustness across diverse challenging conditions. Extensive experiments in the CARLA simulator demonstrate the efficacy of the proposed method, underscoring its potential to enhance machine perception while simultaneously providing interpretable visualizations that support human understanding and decision-making. By integrating weather-invariant image restoration with high-performance detection, our framework offers a practical and effective pathway toward achieving resilient, all-weather perception for safe and reliable autonomous driving.

## 2. Related works

Adverse weather conditions, such as rain, fog, and low-light environments, pose significant challenges to vision-based object detection systems deployed in autonomous vehicles. At the physical level, such conditions substantially impair image fidelity by introducing diverse and scene-dependent visual distortions. For instance, rain introduces high-frequency noise and occlusions through raindrops and streaks on the lens, while fog and haze lead to low-contrast imagery due to light scattering and absorption, which can be modeled using atmospheric scattering models [20,21]. These sensor-level degradations exert a detrimental influence on the reliability and accuracy of subsequent perception algorithms. At the algorithmic level, adverse weather often blurs object contours, diminishes texture and edge fidelity, and weakens semantic cues, thereby compromising feature representation quality and lowering detection confidence [18]. Recent empirical studies have quantified these effects. For example, Mușat et al. [11] reported that heavy rainfall can cause the mean Average Precision (mAP) of state-of-the-art object detectors to drop by more than 30% in urban driving scenes, highlighting the urgent need for models that can generalize beyond ideal visual conditions. These findings underscore the critical importance of addressing weather-induced perception degradation as a prerequisite for achieving reliable and safe autonomous navigation in real-world environments. Research on autonomous driving under adverse weather can be broadly categorized into three directions: enhancing detector robustness, restoring or enhancing degraded images, and integrating restoration with perception tasks.

To mitigate the detrimental effects of adverse weather on object detection, a substantial body of research has focused on enhancing the robustness of detection architectures through direct model-level improvements. Several studies have introduced architectural enhancements to widely adopted detectors such as YOLO and Faster R-CNN, integrating weather-aware components and adaptive attention mechanisms. For example, Chen et al. [22] introduced a domain attention module into Faster R-CNN to dynamically adjust feature extraction based on environmental cues, while Ding et al. [23] designed a cross fusion YOLO (CF-YOLO) with a plug-and-play feature aggregation module to handle unfavorable detection problems of vagueness, distortion and covering of snow. Although these models show improved resilience under known weather conditions, their performance often degrades significantly when encountering unseen or compound weather effects. This limitation arises primarily from their reliance on large-scale, weather-diverse training datasets, the acquisition of which is both resource-intensive and logistically demanding.

To address generalization limitations, recent efforts have turned to multi-task learning and domain adaptation techniques to extract weather-invariant representations. Domain adaptive detectors, such as DA-Faster R-CNN [24] and GPA R-CNN [25] employ adversarial training strategies to align feature distributions between clear and adverse weather domains. Other works such as AOD-net [26] and TogetherNet [27] integrate auxiliary tasks such as image enhancement or semantic segmentation to improve robustness through joint optimization. Fang et al. [28] propose an end-to-end architecture that integrates dehazing with detection. The framework employs an attention fusion module and a self-supervised haze-robust loss, optimized via interval iterative training, and achieves superior detection performance in real-world hazy scenes on benchmarks such as RTTS and VOChaze. However, these approaches often incur substantial computational overhead due to additional loss terms and network branches, and they fail to recover interpretable, human-readable clear-weather imagery, which limits their utility in transparency-critical applications like autonomous driving.

In contrast to model-only robustness strategies, a growing line of research integrates image restoration or enhancement into the detection pipeline to jointly address degradation and recognition under adverse weather. Traditional approaches to adverse weather image enhancement and restoration are primarily based on physical models that explicitly characterize the underlying image degradation mechanisms. The atmospheric scattering model [29,20] forms the theoretical foundation for most dehazing algorithms, characterizing the observed hazy image as a combination of direct attenuation and airlight. He et al.’s seminal work on the Dark Channel Prior (DCP) [30] exploits statistical priors on outdoor haze-free images to estimate transmission maps and recover clear scenes, marking a milestone in single-image dehazing. Similarly, Retinex theory-based methods [31,32] have been widely adopted for low-light enhancement by decomposing images into illumination and reflectance components to correct uneven lighting. However, these physics-based techniques often rely on strong assumptions, such as homogeneous haze distribution or Lambertian surfaces that are violated in dynamic real-world scenarios, leading to suboptimal results under complex weather conditions or heterogeneous illumination. In contrast, recent advances in deep learning, particularly generative adversarial networks (GANs) [33], have demonstrated remarkable advantages for weather-related image restoration. Despite these advances, existing approaches still exhibit several limitations. Physics-based methods rely on strong assumptions and often fail in complex, real-world weather conditions, while GAN-based restoration methods have mainly focused on producing visually plausible images without explicitly evaluating or improving downstream perception tasks such as object detection. To the best of our knowledge, no prior work has proposed a GAN-based image translation framework that simultaneously enhances visual quality and directly improves perception performance under diverse adverse weather conditions. This gap motivates the development of our LP-GAN, which integrates restoration and perception to address both visual degradation and detection challenges in a unified framework.

## 3. Method

This study aims to improve the robustness and accuracy of object detection for autonomous vehicles under adverse weather conditions, while providing interpretable visualizations to support human interpretability and decision-making processes. To this end, a deep learning-based object detection model is presented in Section 3.1, followed by the development of our LP-GAN framework in Section 3.2, which performs image-to-image translation to enable robust object detection under adverse weather conditions.

### 3.1 Object detection in autonomous driving

**Fig 1:**
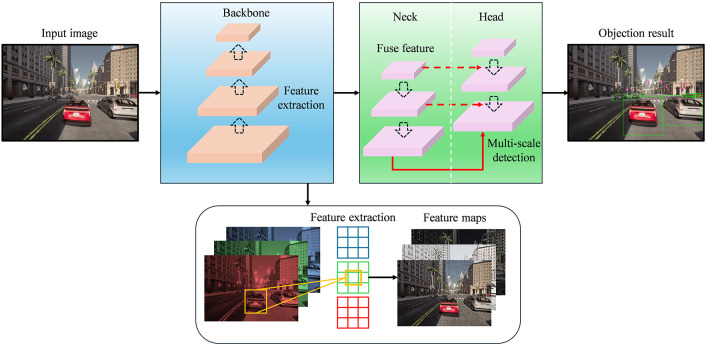
Overview of the YOLOv5 Architecture.

Accurate and real-time object detection is a prerequisite for autonomous driving systems, facilitating safe navigation and autonomous decision-making in complex and dynamic environments. Among the various detection frameworks, the YOLO family has established itself as a highly influential paradigm owing to its optimal trade-off between speed and accuracy. In this work, we adopt YOLOv5, a lightweight yet high-performance object detector, as the core detection backbone, owing to its architectural efficiency and adaptability to embedded automotive platforms. YOLOv5 follows a modular and scalable design, systematically divided into three stages in Fig 1: the Backbone for hierarchical feature extraction, the Neck for multi-scale feature aggregation, and the Head for dense object prediction. Each component is meticulously designed to optimize computational efficiency while preserving fine-grained semantic information, rendering the model well-suited for real-time autonomous driving applications.

The backbone of YOLOv5 serves to extract comprehensive spatial-semantic features from input images. It is based on CSPDarknet, an evolution of Darknet-53, incorporating the Cross Stage Partial Network (CSPNet) architecture [34]. CSPNet is designed to reduce computational overhead while maintaining representational capacity. The core idea is to partition the feature map of the base layer into two parts: one part undergoes a series of convolutional transformations, while the other is bypassed and later fused. This structure promotes gradient propagation and mitigates redundant gradient information, resulting in improved training stability and reduced computational cost in terms of Floating point operations (FLOPs). Each CSP block can be formulated as:


$${𝐅}_{out}=\text{Concat}\left({𝐅}_{bypass},T\left({𝐅}_{transform}\right)\right)$$ (1)

where ${𝐅}_{transform}$ denotes the intermediate features propagated through stacked convolutional layers with non-linear activations, T(·) encapsulates a sequence of transformations comprising convolution, batch normalization, and activation functions such as LeakyReLU, ${𝐅}_{bypass}$ denotes the identity-mapped feature map via a residual shortcut connection, and ${𝐅}_{out}$ is the aggregated output that retains both low-level and high-level representations. The Focus module, unique to YOLOv5, spatially reorganizes the input image $I\in R^{H\times W\times C}$ by slicing and interleaving spatial patches to embed spatial information into the channel dimension:


$${𝐅}_{focus}=\text{Concat}\left(I_{\text{0}::\text{2},\text{0}::\text{2}},I_{\text{1}::\text{2},\text{0}::\text{2}},I_{\text{0}::\text{2},\text{1}::\text{2}},I_{\text{1}::\text{2},\text{1}::\text{2}}\right)$$ (2)

where $I_{a::\text{2},b::\text{2}}$ denotes sub-sampled slices of the input with stride 2 starting from pixel offset (a,b), and ${𝐅}_{focus}\in R^{\frac{H}{\text{2}}\times \frac{W}{\text{2}}\times \text{4}C}$ is the resultant feature tensor.

To preserve multi-scale contextual information, the neck integrates a FPN and a Path Aggregation Network (PANet) [35], facilitating both top-down and bottom-up information flows. The top-down pathway of FPN is expressed as:


$${𝐅}_{l}^{\text{FPN}}=\text{Upsample}\left({𝐅}_{l+\text{1}}\right)+\text{Conv}\left({𝐅}_{l}\right)$$ (3)

and the complementary bottom-up enhancement via PANet is given by:


$${𝐅}_{l}^{\text{PANet}}=\text{Conv}\left({𝐅}_{l-\text{1}}^{\text{FPN}}\right)+\text{Conv}\left({𝐅}_{l}^{\text{FPN}}\right)$$ (4)

where ${𝐅}_{l-\text{1}}$, ${𝐅}_{l}$ and ${𝐅}_{l+\text{1}}$ are the original feature maps from consecutive backbone layers, Conv(·) and Upsample(·) represent a convolutional transformation and an upsampling operation typically implemented via interpolation or transposed convolution. Finally, the detection head produces dense predictions across multiple grid scales, each generating bounding boxes, objectness scores, and class probabilities. The output tensor is structured as $𝐘\in R^{S\times S\times A\times (\text{5}+C)}$, where S denotes the grid size along spatial dimensions, A is the number of anchors per grid cell (typically 3), 5 represents bounding box parameters (x,y,w,h,obj), C is the number of object classes. The bounding box regression is parametrized as:


$$\hat{x}=\sigma (x)+c_{x},\hat{y}=\sigma (y)+c_{y},\hat{w}=p_{w}\cdot e^{w},\hat{h}=p_{h}\cdot e^{h}$$ (5)

where (x,y,w,h) are the raw network outputs, σ(·) is the sigmoid function to constrain coordinates to [0,1], $\left(c_{x},c_{y}\right)$ denote the grid offset corresponding to the location of the prediction cell, $\left(p_{w},p_{h}\right)$ are the anchor dimensions, $\left(\hat{x},\hat{y},\hat{w},\hat{h}\right)$ are the final decoded bounding box parameters in image coordinates. Furthermore, YOLOv5 supports predictions at three different scales (e.g., 80 × 80, 40 × 40, 20 × 20 for an input size of 640 × 640), allowing the network to detect both large and small objects concurrently.

### 3.2 Lightweight Pix2Pix-based generative adversarial network

GANs constitute a prominent class of generative models that learn complex data distributions through adversarial interplay between a generator and a discriminator. Since the seminal work by Goodfellow et al [36], GANs have demonstrated remarkable success in diverse image-to-image translation tasks, including image synthesis, domain adaptation, and image restoration. However, conventional GAN architectures are often ill-suited for structured translation tasks where the output image must preserve spatial alignment with the input, such as weather condition translation for autonomous driving perception. To address this, the Pix2Pix GAN framework introduced by Isola et al. [37] extends the traditional GAN by conditioning both the generator and discriminator on paired input images. This allows the model to learn a direct mapping from a source domain (e.g., foggy, rainy, or nighttime imagery) to a target domain (e.g., clear-weather scenes) while maintaining structural consistency. Specifically, the Pix2Pix architecture employs a U-Net-based generator that leverages skip connections to retain fine-grained spatial details, alongside a PatchGAN discriminator that evaluates local realism over image patches, thereby enhancing texture fidelity. While highly effective, the original Pix2Pix model incurs considerable computational cost due to its dense convolutional layers and symmetric encoder-decoder design, thereby constraining real-time deployment on embedded autonomous driving platforms. In this condition, we propose a LP-GAN tailored for adverse-weather-to-clear-weather image translation with computational efficiency as a central design principle. By introducing architectural simplifications such as depthwise separable convolutions, attention-guided skip connections, and a reduced channel base, we retain the translation fidelity of the original model while dramatically reducing parameter count and inference latency. This lightweight design is particularly advantageous for real-time perception stacks in autonomous vehicles, where low-latency, high-throughput image enhancement is imperative for downstream tasks such as object detection and tracking.

**Fig 2:**
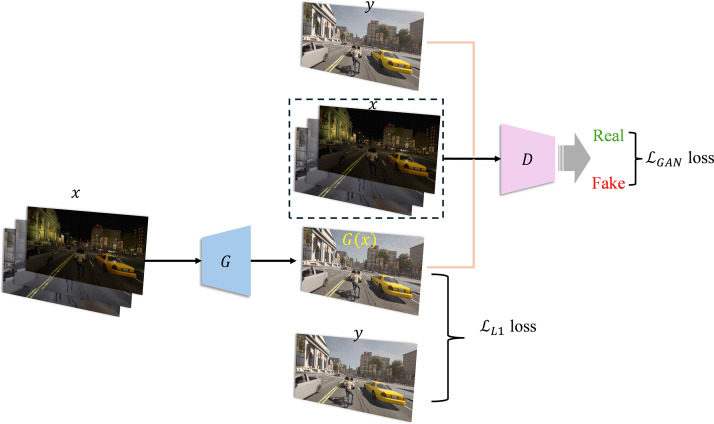
The architecture of the proposed LP-GAN.


The LP-GAN formulates image-to-image translation as a supervised conditional generation task, grounded in a carefully designed objective function that combines adversarial learning with reconstruction fidelity. The architecture of the proposed LP-GAN is shown in Fig 2. Formally, let x∈X denotes an image from the source domain (e.g., rainy or foggy conditions), y∈Y represents its corresponding ground truth image from the target domain (i.e., clear-weather condition). The generator G:X→Y aims to synthesize an output image $\hat{y}=G(x)$ that is both visually realistic and structurally aligned with y, while the discriminator D:(X,Y)→[0,1] seeks to distinguish between real image pairs (x,y) and synthetic pairs (x,G(x)). The full loss function of the LP-GAN is defined as a linear combination of an adversarial loss $L_{GAN}$ and a reconstruction loss $L_{L\text{1}}$, given by

$$L(G,D)=L_{GAN}(G,D)+\lambda L_{L\text{1}}(G)$$ (6)

where λ is a weighting coefficient that balances perceptual realism and pixel-level accuracy. The adversarial loss is formulated as a conditional GAN loss:


$$L_{GAN}(G,D)=E_{x,y}[logD(x,y)]+E_{x}[log(\text{1}-D(x,G(x)))]$$ (7)

which encourages G(x) to be indistinguishable from the real target y under the conditional input x. To enforce low-frequency and structural accuracy, a pixel-wise L1 loss is applied between the synthesized and ground-truth images:


$$L_{L\text{1}}(G)=E_{x,y}\left[∥y-G(x){∥}_{\text{1}}\right]$$ (8)

To enhance computational efficiency and promote deployment feasibility on resource-constrained platforms, LP-GAN leverages depthwise separable convolutions (DSCs) as a principal mechanism for parameter reduction, replacing conventional convolutional layers in both the encoder and decoder pathways shown in Table 1 and 2. Formally, a standard convolution of an input tensor $\text{X}\in R^{H\times W\times C_{in}}$ with $C_{out}$ filters incurs a computational cost of $O\left(K^{\text{2}}C_{in}C_{out}\right)$ where K is the kernel size. In contrast, a depthwise separable convolution decomposes this operation into a depthwise stage of cost $O\left(K^{\text{2}}C_{in}\right)$ and a pointwise stage of cost $O\left(C_{in}C_{out}\right)$, yielding substantial savings in both parameters and FLOPs. Furthermore, the U-Net structure in generator is preserved via symmetric skip connections, enabling the decoder to recover high-resolution spatial details lost during down sampling. For discriminator, we use a non-separable initial convolution to ensure robust low-level feature extraction, and only downsample spatial resolution where necessary, preserving fine-grained details crucial for PatchGAN-style discrimination. These lightweight architectural choices make our discriminator well-suited for real-time applications or deployment in resource-constrained environments without compromising adversarial training stability.

**Table 1:** Architecture of the generator in LP-GAN.

Stage	Layer Type	Kernel Size/ Stride/ Padding	Output Shape	Activation
Input	—	—	3 × 360 × 720	—
Encoder	Depthwise Separable Conv	4 × 4/ 2/ 1	64 × 180 × 360	LeakyReLU
Down1	Depthwise Separable Conv	4 × 4/ 2/ 1	128 × 90 × 180	LeakyReLU
Down2	Depthwise Separable Conv	4 × 4/ 2/ 1	256 × 45 × 90	LeakyReLU
Down3	Depthwise Separable Conv	4 × 4/ 2/ 1	512 × 22 × 45	LeakyReLU
Down4	Depthwise Separable Conv	4 × 4/ 2/ 1	512 × 11 × 22	LeakyReLU
Down5	Depthwise Separable Conv	4 × 4/ 2/ 1	512 × 5 × 11	LeakyReLU
Down6	Depthwise Separable Conv	4 × 4/ 2/ 1	512 × 2 × 5	LeakyReLU
Bottleneck	Depthwise Separable Conv	4 × 4/ 2/ 1	512 × 1 × 2	ReLU
Up1	Depthwise Separable Deconv	4 × 5/ 2/ 1	512 × 2 × 5	ReLU
Up2	Depthwise Separable Deconv	5 × 5/ 2/ 1	512 × 5 × 11	ReLU
Up3	Depthwise Separable Deconv	5 × 4/ 2/ 1	512 × 11 × 22	ReLU
Up4	Depthwise Separable Deconv	4 × 5/ 2/ 1	512 × 22 × 45	ReLU
Up5	Depthwise Separable Deconv	5 × 4/ 2/ 1	256 × 45 × 90	ReLU
Up6	Depthwise Separable Deconv	4 × 4/ 2/ 1	128 × 90 × 180	ReLU
Up7	Depthwise Separable Deconv	4 × 4/ 2/ 1	64 × 180 × 360	ReLU
Output	Depthwise Separable Decon+Tanh	4 × 4/ 2/ 1	3 × 360 × 720	Tanh

**Table 2:** Architecture of the discriminator in LP-GAN.

Stage	Layer Type	Kernel Size/ Stride/ Padding	Output Shape	Activation
Input	Input Image Pair (x, y)	—	6 × 360 × 720	—
Conv1	Conv2d	4 × 4/ 2/ 1	32 × 180 × 360	LeakyReLU (0.2)
Block1	Depthwise Separable Conv	3 × 3/ 2/ 1	64 × 90 × 180	LeakyReLU (0.2)
Block2	Depthwise Separable Conv	3 × 3/ 2/ 1	128 × 45 × 90	LeakyReLU (0.2)
Block3	Depthwise Separable Conv	3 × 3/ 1/ 1	256 × 45 × 90	LeakyReLU (0.2)
Output	Conv2d	4 × 4/ 1/ 1	1 × 44 × 89	—

## 4. Numerical experiments

In this section, we first evaluate the proposed LP-GAN on a large-scale dataset generated using the CARLA simulator, aiming to translate images captured under adverse weather conditions into their corresponding clear-weather counterparts. The translated images are then input into YOLOv5, to assess whether the translation enhances detection robustness and accuracy under challenging environmental conditions.

### 4.1 Evaluation metrics

Evaluating the quality of synthesized images is an open and difficult problem [38]. To quantitatively evaluate the quality of image translation from adverse to clear-weather conditions, we adopt three widely recognized metrics: Peak Signal-to-Noise Ratio (PSNR) [39], Structural Similarity Index Measure (SSIM) [40], and Learned Perceptual Image Patch Similarity (LPIPS) [41]. These metrics jointly capture pixel-level fidelity, structural consistency, and perceptual realism, providing a comprehensive assessment of translation performance.

#### (1) PSNR.

PSNR evaluates the reconstruction quality by measuring the ratio between the maximum possible pixel intensity and the mean squared error (MSE) between the generated image $\hat{I}$ and the reference image I. It is defined as:


$$PSNR=\text{1}\text{0}\cdot {log}_{\text{1}\text{0}}\left(\frac{L^{\text{2}}}{MSE}\right),\;where\text{ MSE}=\frac{\text{1}}{HW}\sum_{i=\text{1}}^{H}\;\sum_{j=\text{1}}^{W}\;{\left(I_{ij}-{\hat{I}}_{ij}\right)}^{\text{2}}$$ (9)

where L denotes the dynamic range of pixel values (e.g., 255 for 8-bit images), H and W are the image height and width. A higher PSNR value indicates better fidelity with respect to the reference image.

#### (2) SSIM.

SSIM aims to quantify image quality degradation based on changes in structural information, luminance, and contrast. It is computed over local image patches and defined as:


$$SSIM(I,\hat{I})=\frac{\left(\text{2}\mu_{I}\mu_{\hat{I}}+C_{\text{1}}\right)\left(\text{2}\sigma_{I\hat{I}}+C_{\text{2}}\right)}{\left(\mu_{I}^{\text{2}}+\mu_{\hat{I}}^{\text{2}}+C_{\text{1}}\right)\left(\sigma_{I}^{\text{2}}+\sigma_{\hat{I}}^{\text{2}}+C_{\text{2}}\right)}$$ (10)

where μ, σ, and $\sigma_{I\hat{I}}$ denote the mean, variance, and covariance of image patches, respectively, and $C_{\text{1}}$ and $C_{\text{2}}$ are small constants to stabilize the division. SSIM values range from 0 to 1, with higher values indicating greater structural similarity.

#### (3) LPIPS.

LPIPS compares deep feature representations extracted from pretrained convolutional neural networks to assess perceptual similarity between images. Given deep features $f_{l}$ at layer l, LPIPS is defined as:


$$LPIPS(I,\hat{I})=\sum_{l}\;\frac{\text{1}}{H_{l}W_{l}}\sum_{h=\text{1}}^{H_{l}}\;\sum_{w=\text{1}}^{W_{l}}\;∥w_{l}⊙\left(f_{l}^{I}(h,w)-f_{l}^{\hat{I}}(h,w)\right){∥}_{\text{2}}^{\text{2}}$$ (11)

where $w_{l}$ is a learned weight vector and ⊙ denotes element-wise multiplication. LPIPS reflects human perceptual judgments more accurately than pixel-wise metrics; lower scores indicate higher perceptual similarity. Together, these metrics form a robust evaluation framework to quantitatively analyze the effectiveness of adverse-to-clear weather image translation in both low-level fidelity and high-level perceptual quality.

To comprehensively evaluate the impact of image translation on downstream object detection performance, we adopt the mean Average Precision (mAP) metric, a widely used standard in the object detection community. The mAP is derived from several fundamental concepts, including Intersection over Union (IoU), Precision, and Recall, and it provides a holistic measurement of detection accuracy across multiple object categories and confidence thresholds. The IoU measures the spatial overlap between a predicted bounding box $B_{p}$ and the ground truth bounding box $B_{gt}$. It is defined as


$$IoU=\frac{\left|B_{p}\cap B_{gt}\right|}{\left|B_{p}\cup B_{gt}\right|}$$ (12)

A prediction is considered a true positive if the IoU exceeds a certain threshold (e.g., 0.5), and otherwise a false positive. Given a set of predicted and ground-truth boxes, the Precision P and Recall *R*

R are defined as:


$$P=\frac{TP}{TP+FP},R=\frac{TP}{TP+FN}$$ (13)

where TP, FP, and FN denote the number of true positives, false positives, and false negatives, respectively. The Average Precision (AP) summarizes the precision-recall curve for a single class by computing the area under the curve:


$$AP=\int_{\text{0}}^{\text{1}}\;P(R)dR$$ (14)

In practice, AP is often approximated by discrete summation over recall levels:


$$AP=\sum_{n=\text{1}}^{N}\;\left(R_{n}-R_{n-\text{1}}\right)\cdot P_{n}$$ (15)

Where $R_{n}$ and $P_{n}$ are the precision and recall at the n-th threshold. The mean Average Precision is computed by averaging the AP scores over all object categories C:


$$mAP=\frac{\text{1}}{\left|C\right|}\sum_{c\in C}\;{AP}_{c}$$ (16)

In our evaluation, we adopt mAP@0.5, where a detection is considered correct if IoU > 0.5, and mAP@[0.5:0.95], which averages AP over multiple IoU thresholds ranging from 0.5 to 0.95 in steps of 0.05, following COCO evaluation standards.

### 4.2 Results on image to image translation

**Fig 3:**
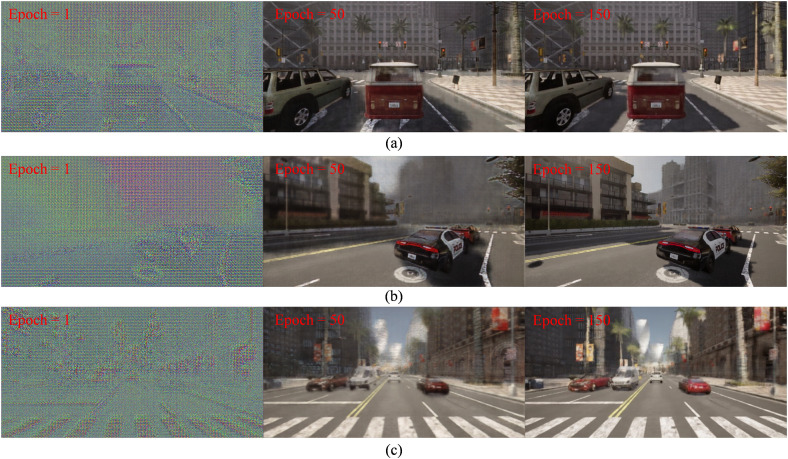
Evolution of generator outputs during training on (a) rainy, (b) foggy and (c) nighttime scenes.

To facilitate the training and evaluation of the proposed LP-GAN framework, we developed a large-scale dataset generated from the CARLA autonomous driving simulator. A total of 40,000 high-resolution RGB images were collected, evenly distributed across four representative weather conditions: clear, rainy, foggy, and nighttime (10,000 images per condition). In the training process, we adopt a batch size of 4, a total of 150 training epochs, a learning rate of $\text{2}\times {\text{1}\text{0}}^{-\text{4}}$, and set the loss balance coefficient λ to 1.5. Fig 3 illustrates the progressive refinement of the generator’s output over the training process. At the initial epoch (epoch = 1), the generator merely captures the coarse spatial layout of the scene. By epoch 50, the generator demonstrates substantial translation capability, effectively mitigating weather-induced degradations such as rain streaks, fog density, and low-light conditions. However, the results remain visually ambiguous in distant regions, with fine structures such as traffic signs, headlight reflections, and background facades appearing overly smoothed or poorly defined, highlighting the model’s current limitations in capturing high-frequency details. After 150 epochs of training, the model demonstrates a notable enhancement in fine-grained realism. The restored images not only preserve global scene geometry but also exhibit significant improvement in reconstructing delicate visual elements, such as traffic light signals, reflective surfaces, and architectural textures at long range. This progression suggests that LP-GAN benefits from a sufficient number of training iterations to stabilize adversarial training dynamics and effectively learn the cross-domain mapping from degraded to clear-weather domains.

**Fig 4:**
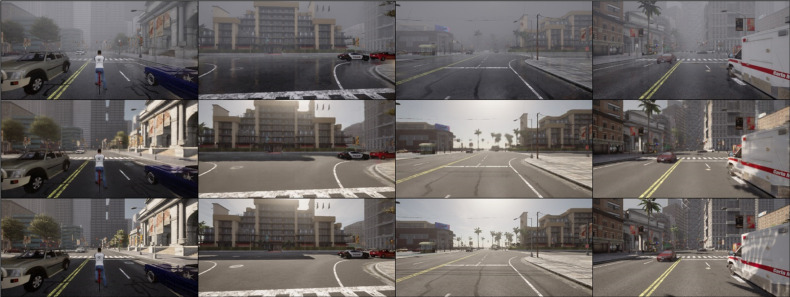
Visual results of rainy-to-clear image translation using LP-GAN, with each column showing (from top to bottom) the rainy input, the generated output, and the clear-weather ground truth.

**Fig 5:**
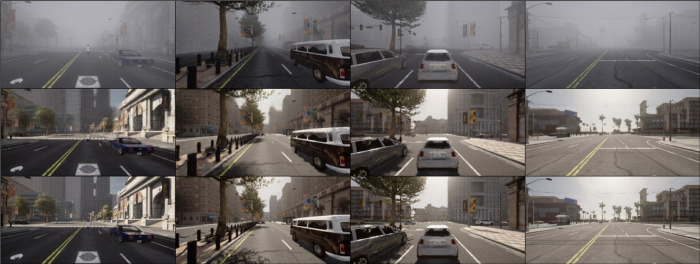
Visual results of foggy-to-clear image translation using LP-GAN, with each column showing (from top to bottom) the foggy input, the generated output, and the clear-weather ground truth.

**Fig 6:**
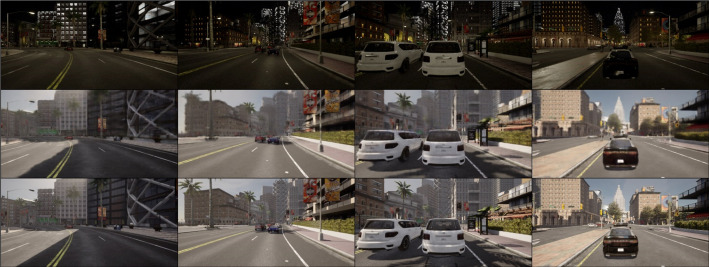
Visual results of nighttime-to-clear image translation using LP-GAN, with each column showing (from top to bottom) the nighttime input, the generated output, and the clear-weather ground truth.

Figs 4-6 present qualitative results of the trained LP-GAN under three representative adverse weather conditions: rain, fog, and nighttime. In the rainy scenario, we simulate a heavy downpour rather than light rain or drizzle. This scenario features prominent rain streaks, water splashes, puddles, and strong surface reflections, which significantly degrade scene visibility. The foggy scenario corresponds to a medium-density ground fog, where distant objects appear blurred and desaturated with reduced contrast and weakened edge information, leading to a marked degradation in scene visibility. The nighttime scenario reflects post-sunset illumination, with significantly reduced overall brightness, deeper shadows, and vehicle headlights and streetlights as the dominant light sources, resulting in low contrast, increased noise, and greater difficulty in perceiving lane markings and object boundaries. As shown in Fig 4, LP-GAN effectively eliminates rain-induced degradations such as atmospheric water streaks and reflective noise on road surfaces. The translated images restore clear sky appearance and road texture continuity, enabling downstream object detectors to identify lane markings and small obstacles more reliably. In Fig 5, under dense fog conditions, the model successfully reconstructs structural details of distant vehicles and buildings that are heavily obscured in the input. Notably, LP-GAN preserves semantic coherence while enhancing visibility in low-contrast regions, which is critical for long-range perception in autonomous navigation tasks. Fig 6 demonstrates the model’s robustness under nighttime scenes, where challenges arise from low illumination and glare. LP-GAN is able to recover ambient lighting, highlight reflective surfaces such as car bodies and signposts, and restore the color consistency of traffic signals. This enhances the visibility of both static infrastructure and dynamic agents, directly benefiting real-time decision-making in urban environments. Overall, the qualitative results demonstrate that LP-GAN generates visually realistic and semantically consistent clear-weather reconstructions across diverse adverse conditions. This capability facilitates improved downstream perception, as detailed subsequently.

**Fig 7:**
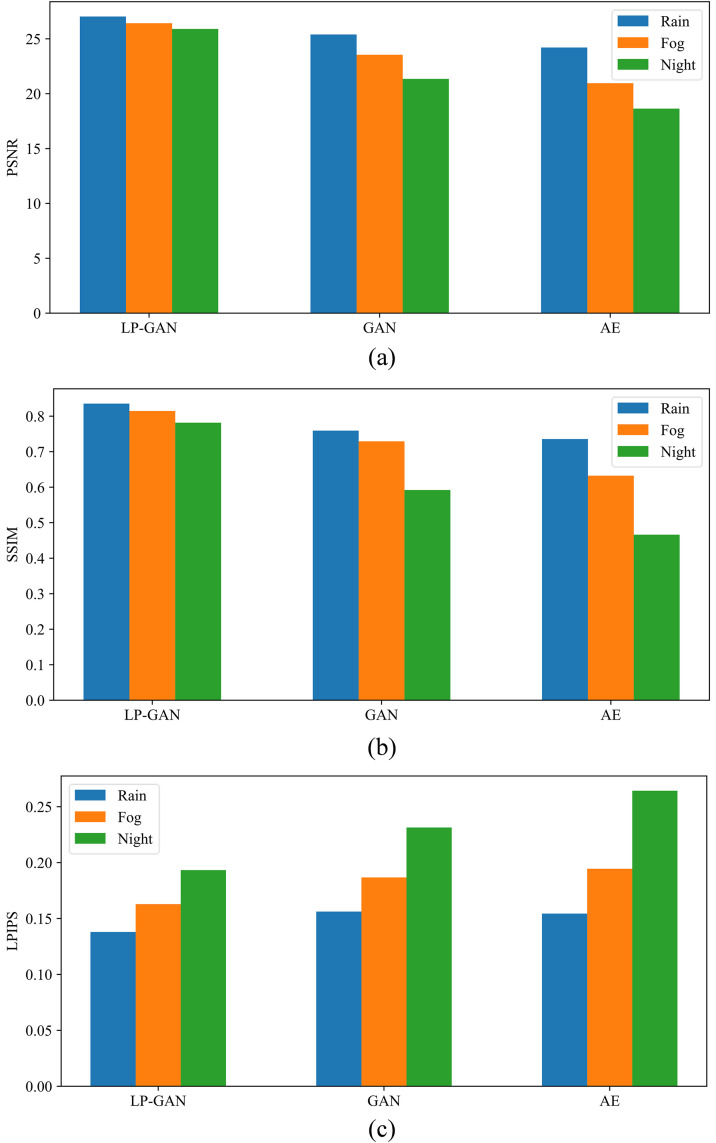
Comparative performance of LP-GAN, standard GAN, and AE on (a) rainy, (b) foggy, and (c) nighttime scenes.

To further validate the effectiveness of our proposed LP-GAN, we conduct a comparative evaluation against two baseline models: a standard GAN and a vanilla autoencoder (AE). As shown in Fig 7, LP-GAN consistently outperforms both baselines across three widely adopted image quality metrics (i.e., PSNR, SSIM, and LPIPS), demonstrating superior fidelity and perceptual realism in translated images.

Across all evaluated metrics, a clear performance ranking emerges, that is, LP-GAN consistently achieves the highest fidelity, followed by the standard GAN, while the autoencoder attains the lowest scores. This is expected, as the autoencoder lacks adversarial training and thus tends to produce overly smoothed outputs with diminished structural detail. Despite leveraging adversarial learning, the standard GAN is not explicitly guided by reconstruction objectives, which can produce textural inconsistencies and spatial distortions. By integrating adversarial loss with pixel-wise and perceptual consistency objectives and employing depthwise separable convolutions for efficient feature modeling, LP-GAN achieves both robust translation fidelity and computational efficiency. Consequently, the hybrid design allows LP-GAN to effectively retain semantic integrity while reconstructing fine-grained image details, even in the presence of severe degradations.

Furthermore, we observe a variation in restoration performance across different weather conditions, generally following the order of rainy, foggy, and nighttime scenarios. This trend reflects the increasing inherent complexity of the degradations associated with each condition. In rainy conditions, high-frequency artifacts such as streaks and reflections are spatially localized, allowing them to be effectively mitigated through targeted filtering operations. Fog, however, causes global contrast attenuation due to light scattering, making it more difficult to recover depth and texture information. Among the conditions considered, nighttime scenes present the most severe challenge, as extreme low-light and pronounced non-uniform illumination markedly impair feature visibility and color fidelity. Collectively, these observations underscore the necessity of developing translation networks capable of adaptively accommodating the heterogeneous characteristics of adverse weather degradations.

### 4.2 Results on objection detection

In this section, we investigate the potential benefits of the proposed LP-GAN in enhancing object detection robustness under adverse weather conditions in autonomous driving. To establish a reliable detection baseline, we first train the YOLOv5 model using a clean subset of our CARLA dataset, which consists exclusively of clear-weather images. The model is trained for 100 epochs using the Adam optimizer with an initial learning rate of $\text{2}\times {\text{1}\text{0}}^{-\text{4}}$, a batch size of 16, and weight decay set to $\text{5}\times {\text{1}\text{0}}^{-\text{5}}$. Cosine annealing is employed to adjust the learning rate dynamically, and data augmentation techniques such as mosaic augmentation, random horizontal flipping, and color jittering are utilized to improve generalization.

**Fig 8:**
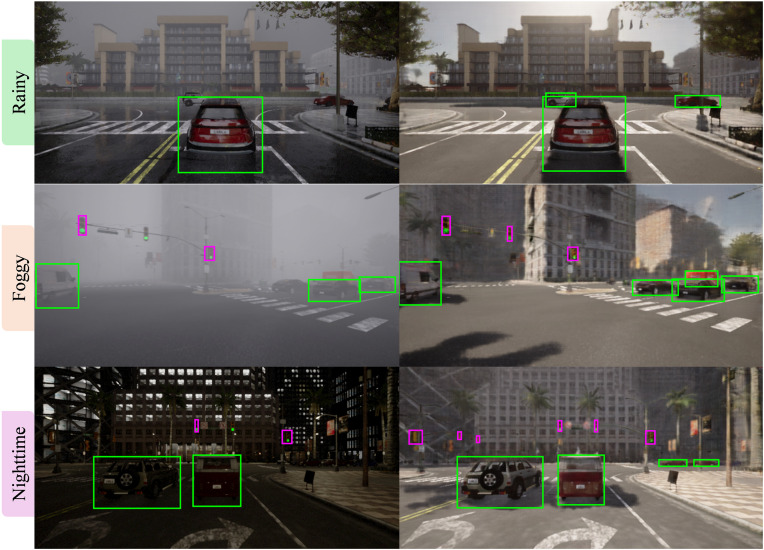
Object detection results in three weather conditions.

Although YOLOv5 demonstrates high accuracy under clear-weather conditions, its performance deteriorates markedly when confronted with rainy, foggy, or nighttime scenarios. As illustrated in Fig 8 (left column), the model frequently fails to detect critical objects such as vehicles and traffic lights in adverse weather environments. The degradation primarily arises from domain shifts induced by weather-specific artifacts, including rain-induced occlusions, fog-related visibility attenuation, and the loss of texture and contrast under low-light nighttime conditions. These phenomena severely impair the model’s ability to extract discriminative features and accurately localize objects. In autonomous driving applications, these missed detections can have severe implications, potentially resulting in failure to yield, misinterpretation of traffic signals, or collisions with previously unrecognized obstacles. To address this challenge, we integrate the LP-GAN module into the perception pipeline. In the proposed pipeline, images acquired under adverse weather are first translated by LP-GAN into photorealistic clear-weather counterparts (Fig 8, right column), which subsequently serve as input to the YOLOv5 detector. This preprocessing step effectively mitigates the domain shift and restores key visual cues, such as the structural integrity of distant vehicles, the visibility of lane markings, and the luminance of traffic signals. As a result, the enhanced pipeline successfully recovers object detection performance under challenging conditions, demonstrating improved detection of both large and small-scale targets. These results underscore the pivotal contribution of generative weather translation toward enhancing perception robustness in real-world autonomous driving environments.

**Fig 9:**
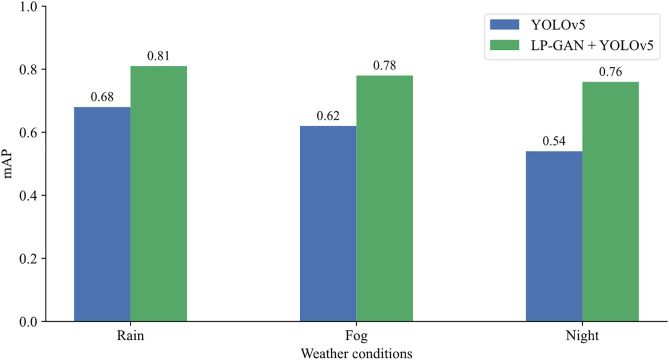
mAP comparison under three different adverse weather conditions.

Quantitative analysis of the object detection performance under different weather conditions is illustrated in Fig 9. Specifically, we compare the mAP of YOLOv5 in two settings: (1) directly applying YOLOv5 to raw adverse-weather images, and (2) applying YOLOv5 to images first translated into clear-weather counterparts by our LP-GAN. For the original adverse-weather images, the mAP values are 0.68 for rainy, 0.62 for foggy, and 0.54 for nighttime scenarios. The comparatively low mAP values underscore the substantial impact of environmental degradations, including rain-induced occlusions, fog-induced visibility attenuation, and nighttime illumination loss. When LP-GAN is employed as a pre-processing module to translate adverse-weather images into clearer views, the detection accuracy improves substantially, reaching 0.81 (rainy), 0.78 (foggy), and 0.76 (nighttime).

The observed gains, exceeding 0.1 mAP across all scenarios, demonstrate the efficacy of the LP-GAN enhancement pipeline in counteracting weather-induced performance degradation. By restoring key visual semantics such as object contours, textures, and illumination cues, LP-GAN enables downstream detectors to operate with enhanced robustness and reliability. This performance improvement is especially significant in the context of autonomous driving, where missed detections of critical objects (e.g., vehicles, pedestrians, or traffic signals) under adverse weather can precipitate hazardous decisions. The proposed LP-GAN framework, by improving perception reliability, contributes to safer and more dependable autonomous navigation. Moreover, from the passenger’s perspective, the image enhancement provided by LP-GAN contributes not only to technical perception improvements but also to the psychological acceptance of autonomous systems. By producing clearer and more interpretable visual outputs even under adverse weather, LP-GAN increases the perceived transparency and reliability of the vehicle’s decision-making process. This, in turn, can significantly reduce passenger anxiety and foster greater trust in the safety and robustness of autonomous driving, especially in challenging real-world scenarios. Nevertheless, detection performance continues to decline from rainy to foggy and nighttime conditions, even with LP-GAN enhancement, highlighting the persistent difficulty of extreme low-light scenarios. Future work may explore the integration of LP-GAN with multimodal inputs (e.g., LiDAR, thermal imaging), temporal consistency modeling, or low-light-specific enhancement techniques to further address these limitations in the most challenging environments.

## 5 Conclusions

In this study, we propose LP-GAN, a lightweight generative adversarial framework tailored for translating adverse weather images under rainy, foggy, and nighttime conditions into their clear-weather counterparts, thereby enhancing the visual input quality for downstream autonomous driving perception tasks. Extensive experiments conducted on a large-scale dataset generated via the CARLA simulator demonstrate that LP-GAN significantly improves image fidelity across multiple perceptual metrics, including PSNR, SSIM, and LPIPS. Furthermore, by integrating LP-GAN with state-of-the-art object detection models such as YOLOv5, we observe consistent improvements in detection accuracy across challenging weather conditions, validating the model’s practical effectiveness in real-world autonomous driving pipelines. In addition to technical benefits, our approach also enhances transparency and interpretability, thereby improving user trust and reducing passenger anxiety in adverse environments. Beyond its experimental validation, LP-GAN demonstrates substantial potential for real-world deployment in both commercial and industrial contexts. In autonomous driving applications, it enhances vehicle safety and reliability by mitigating perception failures caused by adverse weather. Within intelligent transportation systems, LP-GAN can facilitate the development of more resilient infrastructure by providing weather-robust visual inputs. Additionally, it serves as a cost-effective tool for data augmentation in industrial testing pipelines, reducing reliance on labor-intensive and expensive real-world adverse-weather data collection.

Future work will focus on extending LP-GAN to support a broader range of perception tasks, including lane detection, depth estimation, and semantic segmentation, within a unified translation–perception framework. Furthermore, integrating uncertainty quantification into the generative process will enable confidence estimates for translated outputs, which are critical for safe decision-making in autonomous systems. Collectively, these enhancements position LP-GAN to bridge the gap between simulation-based research and large-scale industrial deployment, contributing to the next generation of reliable, trustworthy, and commercially viable autonomous driving technologies.
